# Bibliometrics: Methods for studying academic publishing

**DOI:** 10.1007/s40037-021-00695-4

**Published:** 2021-12-16

**Authors:** Anton Ninkov, Jason R. Frank, Lauren A. Maggio

**Affiliations:** 1 School of Information Studies, University of Ottawa, Ottawa, Ontario Canada; 2 Specialty Education, Royal College of Physicians and Surgeons of Canada, Ottawa, Ontario Canada; 3 Department of Emergency Medicine, University of Ottawa, Ottawa, Ontario Canada; 4 Center for Health Professions Education and Department of Medicine, Uniformed Services University, Bethesda, MD USA

**Keywords:** Bibliometrics, Scholarly communication, Information science

## Abstract

Bibliometrics is the study of academic publishing that uses statistics to describe publishing trends and to highlight relationships between published works. Likened to epidemiology, researchers seek to answer questions about a field based on data about publications (e.g., authors, topics, funding) in the same way that an epidemiologist queries patient data to understand the health of a population. In this Eye Opener, the authors introduce bibliometrics and define its key terminology and concepts, including relational and evaluative bibliometrics. Readers are introduced to common bibliometric methods and their related strengths and weaknesses. The authors provide examples of bibliometrics applied in health professions education and propose potential future research directions. Health professions educators are consumers of bibliometric reports and can adopt its methodologies for future studies.

## Introduction

Have you ever wondered: How can I find collaborators to study the teaching of clinical reasoning? How often do health professions education (HPE) articles draw on studies from the field of education? How can I conduct a systematic review in HPE without knowing which articles or journals “count” as being HPE? Bibliometrics can help answer these questions and more. In this Eye Opener, we describe the background of bibliometrics, highlight the methods bibliometricians use, and discuss how health professions educators have and can apply them in HPE.

## Background

Bibliometrics is the analysis of published information (e.g., books, journal articles, datasets, blogs) and its related metadata (e.g., abstracts, keywords, citations) using statistics to describe or show relationships between published works [1]. We provide key definitions of bibliometric concepts in Tab. 1. Bibliometrics is based on the assumption that a field’s scholarly output is captured in the published literature. To frame this in a medical context, Lewison aptly wrote: *“Bibliometrics is to scientific papers as epidemiology is to patients”* [2]. In parallel to epidemiology, bibliometrics researchers can trace the trajectory of a topic by tracking its spread across the literature or they can determine the characteristics of journal articles that are feverishly downloaded to ascertain impact. Bibliometrics and epidemiology also share approaches in their use of rigorous statistics, conduct of cross-sectional studies, and attempts to identify correlation.

**Table 1:** Key definitions of bibliometric concepts

Term	Definition
Bibliometrics	The analysis of published information (e.g., books, journal articles, datasets, blogs) and its related metadata (e.g., abstracts, keywords, citations) using statistics to describe or show relationships between published work [1]
Evaluative bibliometrics	An approach to bibliometrics that aids in the evaluation of units of analysis [3]
Relational bibliometrics	An approach to bibliometrics that provides insights into the relationships between units of analysis [3]
Metadata	General definition: Data about data. More specific definition: The “internal and external documentation and other data necessary for the identification, representation, interoperability, technical management, performance and use of data contained in an information system” [4]
Impact factor	Used to describe both journals or authors, an impact factor is a representation of the number of citations as a comparison to the number of publications [5]
*h*-Index	The *h*-index is defined as *h* number of papers with at least *h* number of citations. For example, a researcher scientist with an *h*-index of 15 has published 15 papers which have received at least 15 citations each [6]

James McKeen Cattell, editor of *Science* from 1895–1944, is credited as the founder of the systematic collection of statistics on science, which gave birth to the field of bibliometrics [7]. Since then, bibliometricians have expanded the field, conducting important work such as developing and evaluating indicators for research impact (e.g., journal impact factor, *h*-index) and utilizing methods of data visualization to see the relationships between researchers or research items. While bibliometrics developed within the broader field of information science, the methods have increasingly been adopted in and applied to a variety of other disciplines—including HPE.

## Bibliometrics methods

Researchers use a variety of bibliometric methods, which can be generally divided into two categories: *evaluative* and *relational*.* Evaluative bibliometrics* are used to describe the characteristics of published information. For example, if a researcher wants to answer the above question about finding clinical reasoning researchers or to identify the evolution of a topic, they would use *evaluative bibliometrics*. The journal impact factor (JIF), along with other types of impact factors (e.g., *h*-index) are indicators that rely on *evaluative bibliometrics* to articulate the impact of a research output. Based on measurements including the number of citations or publications, these impact metrics provide the means to quickly evaluate scholarly work—albeit with the caveat that these indicators have important limitations that must be considered before applying them in practice (see the Discussion for more on this).

In HPE, *evaluative bibliometrics* have been utilized, most commonly in the form of systematic reviews and meta-analyses, with over 400 published in HPE in the last twenty years [8]. However, HPE researchers have also adopted *evaluative bibliometrics* to answer a variety of questions. For example, *evaluative bibliometrics* was used by HPE researchers to determine how long it takes for a submitted manuscript to be published [9]. To answer this question, they downloaded from PubMed the submission and publication dates for nearly 20,000 HPE articles and computed that the average time from submission to publication was 180.93 days. Madden and colleagues provide another example in their study to understand the proportion of male and female authors across authorship positions in HPE journals in which they examined the author metadata from over 5000 authors [10]. HPE researchers have also employed bibliometrics as a component of studies that use multiple methods. For example, in a survey study researchers crafted their sample by using *evaluative bibliometrics* to extract the first authors of HPE articles published in the previous two years [11].

*Relational bibliometrics *provides *“an overview of the relationships between different actors”* [12]. The idea behind *relational bibliometrics* is that within the metadata collected on various entities (e.g., authors, papers, journals), hidden associations can be identified that facilitate understanding the set of entities at a broader level. Researchers conduct *relational bibliometrics* by investigating the entities’ shared metadata occurrences (e.g., citations, keywords, authors)—the more entities share metadata, the more likely they are similar in some way. If a set of articles or authors all cite the same article or group of articles, it would be possible to infer that the articles or authors are related in some way. For example, to answer the question posed in the introduction, regarding whether or not HPE authors reference the education literature, a researcher could use bibliometric coupling. This method enables researchers to examine a corpus of articles and the references that they cite. The researcher would then be able to investigate whether the cited references were published in higher education journals.

Based on our review of the literature, there are fewer examples of *relational bibliometrics* studies in HPE than those using *evaluative bibliometrics*. However, increasingly researchers have used network analysis in relation to publications and their related metadata [3]. In network analyses, nodes of a network (e.g., group of authors, journals, or institutions) are tied to each other based on various relationships they have (e.g., shared citations, authors, or keywords). By plotting the nodes of the network based on these relationships, one can examine their hidden structures [13, 14]. For example, Peterson et al. examined an HPE research group’s productivity in relation to their external collaborations concluding that increased collaboration translated into rising productivity [15]. In another example, Young and colleagues investigate topics and trends in HPE using network analysis [16]. This limited use of *relational bibliometrics* signals an area ripe for future research in HPE.

## Discussion

Bibliometrics can impact everyone involved in HPE. By characterizing the scholarly output of HPE, bibliometrics can be used to define our field by identifying relevant journals, articles, authors, and topics. Understanding the scope of the field gives us the ability to track trends and identify gaps, but it can also confer a sense of membership to individuals that identify with the field. In HPE, which includes researchers from a variety of training backgrounds and knowledge traditions, identifying our community and its scholarship can be difficult with researchers proposing variable approaches. For example, Lee et al. attempted to define the field of medical education based on a corpus of publications retrieved when searching for “medical education” in MEDLINE [17], whereas other authors propose the use of a core set of journals based on journal impact factor [18] and presence in Web of Science [8]. This has implications for the field’s ability to build on previous scholarship and even apply for funding, suggesting now is the time for medical education to leverage the power of bibliometrics.

The future of bibliometrics is bright, and there are many opportunities for HPE scholars to incorporate bibliometrics into their research. There are many bibliometric studies that could be reproduced in HPE. Collaborators are key for bibliometric studies and adding a bibliometrician or information scientist to the author team would be incredibly valuable for their expertise in information structure and data management. Moreover, these professionals keep abreast of what is happening in bibliometrics. For example, natural language processing tools have been developed to enable researchers to automatically identify the topics in a field of study (e.g., HPE) as well as deploy such approaches in extracting data for knowledge syntheses. To connect with a bibliometrician, consider reviewing faculty biographies on information school websites or reaching out to professional associations such as the Association of Information Science and Technology.

To expand on bibliometrics, there are also emerging mixed methods opportunities that combine with qualitative methods to create a more holistic and accurate picture of scholarly communication beyond academic publishing. These methods have been coined as webometrics (analyzing the reach/impact of web content using adapted methods from bibliometrics) [19] and altmetrics (analyzing academic publishing using non-traditional metadata such as social media reach) [20]. With the technological developments of analyzing and managing big data, the potential applications of bibliometrics and related fields to HPE are immense.

While bibliometrics can be helpful, researchers must also consider its limitations [21]. When applied to analyzing a field like HPE, the accuracy of the metadata for which researchers have access is a key factor. When researchers assign value to certain publishing attributes such as citations, number of publications, or impact factors, incentives arise for researchers to publish more or in specific venues. Furthermore, there is a tendency to compare different fields of research to one another using these metrics, which can be fraught. As a result, the metrics we use to study publication habits or identify prominent researchers or institutions can become less reliable or meaningful [22]. This limitation has been well documented, in particular when it comes to metrics for measuring research output like the *h*-Index or journal impact factor [6, 23, 24].

Bibliometrics and the relevant methods for analyzing scholarly publications have been applied to HPE, but there are enormous opportunities for further development. New ways of analyzing metadata about publishing in academic medicine, especially using *relational bibliometrics* methods, could be helpful for delineating HPE and understanding where cross-disciplinary work is happening. In addition, adopting standards to record metadata for publications and identifying authors (e.g., ORCID) would provide greater ability to analyze and understand HPE as a field. Just as epidemiology is an essential fundamental science for the practice of medicine or nursing, health professions educators should consider adding bibliometrics to their toolkit to understand the scholarship of HPE.
